# ﻿*Fargesianana* (Poaceae, Bambusoideae), a new bamboo species from Yunnan, China

**DOI:** 10.3897/phytokeys.257.154092

**Published:** 2025-06-11

**Authors:** Jian-Wei Li, Juan Wang, Ming Shi, Fan Du

**Affiliations:** 1 College of Forestry, Southwest Forestry University, Kunming, Yunnan 650224, China; 2 Institute of Bamboo and Rattan, Southwest Forestry University, Kunming, Yunnan 650224, China; 3 Forestry and grassland Bureau of Mengla County, Xishuangbanna, Yunnan 666300, China

**Keywords:** Bambusoideae, *Fargesia*, new species, Yunnan

## Abstract

*Fargesianana* (Bambusoideae), a new bamboo species is described and illustrated from Wuding County, Yunnan, China. Based on careful comparison of morphological features and molecular phylogeny evidence, we confirmed its identity as a new member of the genus *Fargesia*. The morphology of this new species is similar to *F.fractiflexa* and *Yushaniapolytricha*, but differs in its ratio of culm leaf sheath length to internode length, shape of blade, foliage leaf dimensions, and distinct transverse veins. Hitherto, this new species is the smallest of *Fargesia* species found and recorded. According to the IUCN Red List Categories and Criteria (IUCN 2024), it meets the criteria to be listed as Critically Endangered (CR).

## ﻿Introduction

*Fargesia* Franch. (1893) was established by the French botanist Adrien M. Franchet in 1893. It is one of the largest genus of the tribe Arundinarieae (Gramineae, Bambusoideae), and consists of shrubby bamboos mainly distributed in subalpine and alpine areas of subtropical Asia (Sun et al. 2016). Until 2008, *Fargesia* contained more than 100 species, and 101 species were found in China, 54 species recorded in Yunnan Province (Yi et al. 2008; Sun et al. 2016). Since 2008, seven new species of *Fargesia* have been published (Yang et al. 2011; Yang and Yi 2013a, 2013b; Sun et al. 2016; Ye et al. 2020; Zhang et al. 2020), and six species were known from China, including five species from Yunnan Province.

Species of *Fargesia* are characterized by pachymorph rhizome with short neck, culms unicaespitose and basally erect, 7–15 branches per node, inflorescence racemose to paniculate, compressed or open, with three stamens (Li et al. 2006). Although reproductive organs are important for bamboos classification, many new species of *Fargesia* have been described and published based on vegetative morphological characters due to the rare flowering nature (Yi et al. 2005, 2006, 2007; Yang et al. 2011; Yang and Yi 2013a, 2013b; Ye et al. 2020; Zhang et al. 2020). Only 19 species have inflorescence information in “Flora Reipublicae Popularis Sinicae” (Keng and Wang 1996) and “Flora of China” (Li et al. 2006). In recent years, with the rapid development of molecular technology, many studies have shown that the reconstruction of molecular phylogenetic trees has played an important role in the confirmation of bamboo new species (Qin et al. 2019; Tong et al. 2020; Wang et al. 2023; Zhang et al. 2024).

During a botanical survey to the Jinsha River basin in November 2020, we collected bamboo specimens in the Jiyi Rift Valley in Jiyi Town, Wuding County, Yunnan Province, China, and suspected it to be a new species. In order to know more about it, we revisited Jiyi Rift Valley at the end of September 2021 and more specimens were collected. This bamboo species has short-necked pachymorph rhizomes, unicaespitose culms with base erect, multiple branches without obvious main branches at the node. These characteristics are typical of *Fargesia*. After comparison with species of *Fargesia* and literature survey (e. g., Keng and Wang 1996; Yi et al. 2005, 2006, 2007, 2008; Li et al. 2006; Yang et al. 2011; Yang and Yi 2013a, 2013b; Ye et al. 2020; Zhang et al. 2020), we found that the morphological characters did not correspond to the available records and descriptions of *Fargesia*, and it was most similar to *F.fractiflexa* and *Yushaniapolytricha*. Accordingly, we described it as a new species of *Fargesia* in this paper.

## ﻿Materials and methods

### ﻿Field investigation and morphological comparison

Specimens were collected in Wuding County in November 2020 and September 2021. At the same time, we also collected young and healthy leaves and dried them in silica gel for the molecular experiments. Morphological features of the new species were observed and measured based on living plants in the field and specimens in the lab. Morphological characters were measured with a ruler, or observed by stereomicroscope. Morphological characteristics of similar species (*F.fractiflexa* T. P. Yi and *Yushaniapolytricha* J. R. Xue & T. P. Yi) were obtained from literature (e.g., Keng and Wang 1996; Li et al. 2006; Yi et al. 2008).

### ﻿DNA extraction, sequencing, and assembly

Total genomic DNA was isolated from foliage leaves dried in silica gel by the TIANGEN Magnetic Plant Genomic DNA Kit (TIANGEN, Beijing, China). All procedures were carried out in accordance with the manufacturer’s instructions. DNA concentration, integrity and purity were assessed using the Agilent 5400 system. Then, the genomic DNA was randomly sheared into fragments of approximately 350 bp using a Covaris Focused-ultrasonicator. Subsequently, the DNA fragments were end-polished reactions at the 3’ and 5’ ends, added A-tail at the 3’, and ligated with the full-length adapter for Illumina sequencing. The DNA fragment size selection was performed by the Agencourt SPR Iselect beads (Beckman Coulter, USA, Catalog #: 2358413), followed by PCR amplification. Library quality was assessed using the Agilent 5400 system (AATI) and precisely quantified effective library concentration by qPCR (1.5 nM). The qualified library data were sequenced on the Illumina Novaseq platform with PE150 method. Finally, approximately 2 GB of data was obtained per sample. All sequencing experiments were performed at Novogene Bioinformatics Technology Co. Ltd. (Beijing, China).

After obtaining the sequencing data in FASTQ format, quality control of the raw data was performed using Fastp (Chen et al. 2018). The plastome and nrDNA sequences were assembled with GetOrganelle (Jin et al. 2020) using default parameters. Subsequently, the assembly graph was visualized using Bandage (Wick et al. 2015) to display the complete plastomes. The assembled plastome sequences were validated by collinear alignment with Mauve (Darling et al. 2004) under default settings, ensuring structural and orientational consistency with the reference plastome sequences. Finally, one of the inverted repeats (IRs) was removed to obtain the plastid sequence. Based on the annotation information of *Fargesiayunnanensis* J. R. Xue & T. P. Yi (Lv et al. 2023), plastid gene annotation was performed using the online tool CPGAVAS2 (Shi et al. 2019), followed by manual adjustments in Geneious Prime (Kearse et al. 2012). Additionally, using the ribosomal DNA (rDNA) sequences of *Yushaniadezhui* Y. X. Zhang & R. L. Zhang (Zhang et al. 2024) as reference, the complete nuclear ribosomal DNA (nrDNA) sequences were assembled and annotated in Geneious Prime 2022.0.1 (Kearse et al. 2012).

### ﻿Phylogenetic tree reconstruction

To determine the phylogenetic position of the new species, we reconstructed phylogenetic trees based on chloroplast datasets using Maximum Likelihood (ML) and Bayesian Inference (BI) approaches. Chloroplast genome sequences and nrDNA sequences of related species were retrieved from previous studies (e.g., Guo et al. 2021; Lv et al. 2023; Zhang et al. 2024) or downloaded from the NCBI database, resulting in 38 sequences from 36 species (Table 1). Multiple sequence alignment was performed using MAFFT (Katoh et al. 2002).

**Table 1.** Voucher information and GenBank accession numbers for plant materials used in this study.

Taxon	Voucher information	GenBank accession No. / source	
		Plastome	nrDNA
**Ingroup**			
*Acidosasapurpurea* (J. R. Xue & T. P. Yi) P. C. Keng	NZY041	NCBI(HQ337793)	NCBI(ON204022)
*Ampelocalamusactinotrichus* (Merr. & Chun) S. L. Chen, T. H. Wen & G. Y. Sheng	ZXZ151102	Guo et al. (2021)	Guo et al. (2021)
*Ampelocalamusluodianensis* T. P. Yi & R. S. Wang	10052	Guo et al. (2021)	Guo et al. (2021)
*Chimonobambusapurpurea* J. R. Xue & T. P. Yi	NH004	NCBI(MW030500)	NCBI(ON203966)
Chimonocalamusdumosusvar.pygmaeus J. R. Xue & T. P. Yi	GC140	Guo et al. (2021)	Guo et al. (2021)
*Chimonocalamuspallens* J. R. Xue & T. P. Yi	13048	Guo et al. (2021)	Guo et al. (2021)
*Fargesiaacuticontracta* T. P. Yi	YXY266-1	Guo et al. (2021)	Guo et al. (2021)
*Fargesiafrigidis* T. P. Yi	ZXZ11023	Guo et al. (2021)	Guo et al. (2021)
*Fargesiaglabrifolia* T. P. Yi	YXY251A	Lv et al. (2023)	Lv et al. (2023)
*Fargesiaglabrifolia* T. P. Yi	YXY251B	Lv et al. (2023)	Lv et al. (2023)
*Fargesiamelanostachys* (Hand.-Mazz.) T. P. Yi	YXY145-3	Guo et al. (2021)	Guo et al. (2021)
*Fargesianivalis* T.P.Yi & J.Y.Shi	YXY125-2	Guo et al. (2021)	Guo et al. (2021)
*Fargesianana* F. Du, M. Shi & J. W. Li	JY0101	NCBI(PV367265)	NCBI(PQ887789)
*Fargesianana* F. Du, M. Shi & J. W. Li	JY0201	NCBI(PV367266)	NCBI(PQ887790)
*Fargesiayunnanensis* J. R. Xue & T. P. Yi	CZM027	Lv et al. (2023)	Lv et al. (2023)
*Fargesiayunnanensis* J. R. Xue & T. P. Yi	CZM028	Lv et al. (2023)	Lv et al. (2023)
*Gaoligongshaniamegalothyrsa* (Hand.-Mazz.) D. Z. Li	GC120-5	Guo et al. (2021)	Guo et al. (2021)
*Gelidocalamuslatifolius* Q. H. Dai & T. C. Chen	GC90-6	Guo et al. (2021)	Guo et al. (2021)
*Hsuehochloacalcarea* (C.D.Chu & C.S.Chao) D.Z.Li & Y.X.Zhang	GC82	Guo et al. (2021)	Guo et al. (2021)
*Indocalamuslatifolius* (Keng) McClure	GC58-2	Guo et al. (2021)	Guo et al. (2021)
*Indosasacrassiflora* McClure	GY15039-B	Guo et al. (2021)	Guo et al. (2021)
*Oligostachyumsulcatum* Z. P. Wang & G. H. Ye	GY15022-F	Guo et al. (2021)	Guo et al. (2021)
*Phyllostachysincarnata* T. W. Wen	ZLN-2011035	Guo et al. (2021)	Guo et al. (2021)
*Pleioblastusfortunei* (Van Houtte ex Munro) Nakai	GC33-2	Guo et al. (2021)	Guo et al. (2021)
*Pseudosasaguanxianensis* T. P. Yi	GC62-3	Guo et al. (2021)	Guo et al. (2021)
*Sasahubeiensis* (C. H. Hu) C. H. Hu	GY15073-B	Guo et al. (2021)	Guo et al. (2021)
Sasaellahidaensisvar.muraii (Makino & Uchida) Sad.Suzuki	GZH-081	Guo et al. (2021)	Guo et al. (2021)
*Semiarundinariaokuboi* Makino	GZH-088	Guo et al. (2021)	Guo et al. (2021)
*Shibataeachinensis* Nakai	GC34-2	Guo et al. (2021)	Guo et al. (2021)
Shibataeananpingensisvar.fujianica (Z. D. Zhu & H. Y. Zhou) C. H. Hu	GC115-1	Guo et al. (2021)	Guo et al. (2021)
Sinobambusatootsikvar.maeshimana Muroi ex Sugimoto	GY15061-D	Guo et al. (2021)	Guo et al. (2021)
*Yushaniabrevipaniculata* (Hand.-Mazz.) T. P. Yi	YXY043	Guo et al. (2021)	Guo et al. (2021)
*Yushanialongiuscula* T. P. Yi	YXY154-1	Guo et al. (2021)	Guo et al. (2021)
*Yushaniamaculata* T. P. Yi	DSTQ01	Zhang et al. (2024)	Zhang et al. (2024)
*Yushanianiitakayamensis* (Hayata) P. C. Keng	12321	Guo et al. (2021)	Guo et al. (2021)
*Yushaniapolytricha* J. R. Xue & T. P. Yi	QZS001	Zhang et al. (2024)	Zhang et al. (2024)
*Yushaniashuichengensis* T.P. Yi & L. Yang	LPS15	Zhang et al. (2024)	Zhang et al. (2024)
**Outgroup**			
*Chusqueaculeou* É.Desv.	GZH-089	Guo et al. (2021)	Guo et al. (2021)

Based on the Bayesian Information Criterion (BIC), the TPM3+F+I+G4 model for plastid genomes and the TPM3+F+I+G4 model for nrDNA were selected using ModelFinder 2.2.5 (Kalyaanamoorthy et al. 2017). Subsequently, maximum likelihood (ML) analysis was performed with IQ-TREE 2.2.5 (Nguyen et al. 2015), employing 1000 ultrafast bootstrap replicates and SH-aLRT tests (Hong et al. 2022). Bayesian Inference (BI) was conducted using MrBayes 3.2.7a (Ronquist et al. 2012), with the GTR+I+G model selected via BIC in jModelTest 2.1.7 (Darriba et al. 2012). Markov Chain Monte Carlo (MCMC) simulations were run for 1,000,000 generations, sampling every 1000 generations, with the first 25% of iterations discarded as burn-in. A 50% majority-rule consensus tree was constructed when the average standard deviation of split frequencies fell below 0.01. Posterior probabilities for each branch were calculated to assess support (Ronquist et al. 2012).

Furthermore, to assess whether different datasets yield consistent phylogenetic topologies, we conducted phylogenetic analyses on both the coding regions (Coding DNA Sequences, CDS) and non-coding intergenic spacer regions (Intergenic Spacers, IGS) of chloroplast genomes. Following the aforementioned methodology, phylogenetic trees were constructed separately using Maximum Likelihood (ML) and Bayesian Inference (BI) approaches (Suppl. material 1).

## ﻿Results

### ﻿Morphological analysis

The new species shares some morphological similarities with *Fargesiafractiflexa* and *Yushaniapolytricha*, but can be distinctly differentiated by the following key characteristics: (1) Compared to *F.fractiflexa*, although both share similar rhizome and culm heights, the new species is distinguishable by the dimensions of its blade size (0.4~2 cm × 0.8~1.7 cm vs. 2~8 cm vs. 1~1.5 mm), foliage leaf size (5~7 cm × 5~7.5 mm vs. 7~13 cm vs. 0.5~1.2 cm); (2) Compared to *Y.polytricha*, while both possess slender rhizomes and relatively short culms, the new species exhibits stable distinctions in shortened culm neck (4 8 cm vs. 13~40 cm), number of branch per node (10~20(30) vs. 1~5), absence of auricles (absent vs. falcate auricle), and foliage leaf sheath length (1.8~2.2 cm vs. 5.5~10 cm). Additionally, the new species is characterized by a unique combination of traits, including its ratio of culm leaf sheath / internode (1/3–1/2 vs. longer than internodes in *F.fractiflexa*, vs. 2/3 in *Y.polytricha*), foliage leaf size(5~7 cm vs. 7~13 cm in *F.fractiflexa*, vs. 9~21 cm in *Y.polytricha*), and transverse veins on foliage leaf blade (conspicuous vs obscure in *F.fractiflexa* and *Y.polytricha*). These features collectively establish its independent taxonomic status and distinguish it from closely related species (see Table 2 for details).

**Table 2.** Morphological comparison of *Fargesianana*, *F.fractiflexa* and *Yushaniapolytricha*.

Characters	*F.nana*	*F.fractiflexa*	*Yushaniapolytricha*
Rhizome	culm-neck 4~8 cm long, 0.6~0.8 cm in diameter	culm-neck 3~20 cm long, 0.7~2 cm in diameter	culm-neck 13~40 cm long, 0.3~0.8 cm in diameter
Culm height	1.5~2.2 m	2~3(4.5) m	1~2 m
Culm diameter	0.2~0.4(0.6) cm	0.6~1.2 cm	0.3~0.8 cm
Internode length	10~19(22) cm	12~15(20) cm	13~25(37) cm
Branch complement	10~20(30)	5~17	1~5
Culm bud	semi-orbicular	semi-orbicular	long-ovate
Culm leaf sheath	caducous, papery, 1/3 to 1/2 as long as internodes	caducous or persistent, thinly coriaceous, longer than internodes	caducous, cartilaginous, 2/3 as long as internodes
Culm leaf ligule length	ca. 2 mm	ca. 1~3 mm	ca. 1 mm
Culm blade size	Linear-triangular, 0.4~2 cm long, 0.8~1.7 cm wide	Linear-lanceolate, 2~8 cm long, 1~1.5 mm wide	Linear-lanceolate, 1~3 cm long, ca. 2 mm wide
Auricle	Absent	Absent	Falcate auricles with margins
Foliage leaf size	length 5~7 cm, width 5~7.5 mm, sparsely gray-pilose upper surface when young	length 7~13 cm, width 0.5~1.2 cm, glabrous	length 9~21 cm, width 1.2~2.5 cm, abaxially grayish-white pubescent.
Foliage leaf sheath length	length 1.8~2.2 cm	length 2~3 cm	length 5.5~10 cm
Foliage leaf ligule length	ca. 1 mm	ca. 1~1.5 mm	ca. 1 mm
Petiole length	ca. 2~3 mm	ca. 1~2 mm	ca. 3~4 mm
Transverse veins on foliage leaf blade	Conspicuous	obscure	obscure
Distribution	Yunnan: Wuding county, Jiyi town, 1895~1926 m	Southwestern Sichuan, northeastern to northwestern Yunnan, 1380~3200 m	Central to western Yunnan, 1900~1950 m

### ﻿Phylogenetic analysis

**Figure 1.**
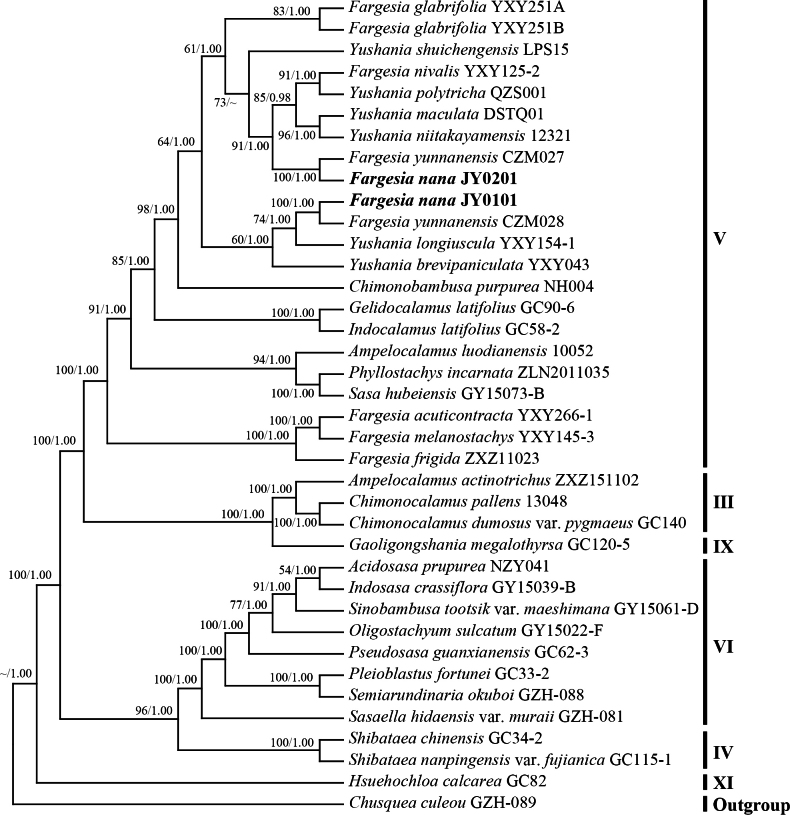
Phylogenetic tree reconstructed by Maximum Likelihood (ML) and Bayesian Inference (BI) analysis based on plastome sequences. Numbers along branches indicate the Maximum Likelihood bootstrap values (MLBP) (left) and Bayesian posterior probabilities (BI) (right). “~”: nodes with Maximum Likelihood bootstrap values (MLBP) <50% (left), Bayesian posterior probabilities (BI) <95% (right). The Roman numbers on the right of this tree correspond to those lineages recovered in previous studies (Yang et al. 2013).

Upon alignment, the total length of the plastome sequence was 143653 bp, including 4325 variable sites and 957 parsimony information sites. In the plastome phylogenetic tree, six clades were recovered, i.e. clades III~VI, clade IX and clade XI. The samples of the new species were located in V clade and formed two subclades. One subclade included JY0201 (MLBP/BI = 100/1.00) and one subclade included JY0101 (MLBP/BI = 100/1.00). The new species was closely related to *F.nivalis*, *Yushaniapolytricha*, *Y.maculata*, *Y.niitakayamensis*, *F.yunnanensis*, and *Y.brevipaniculata* (Fig. 1).

**Figure 2.**
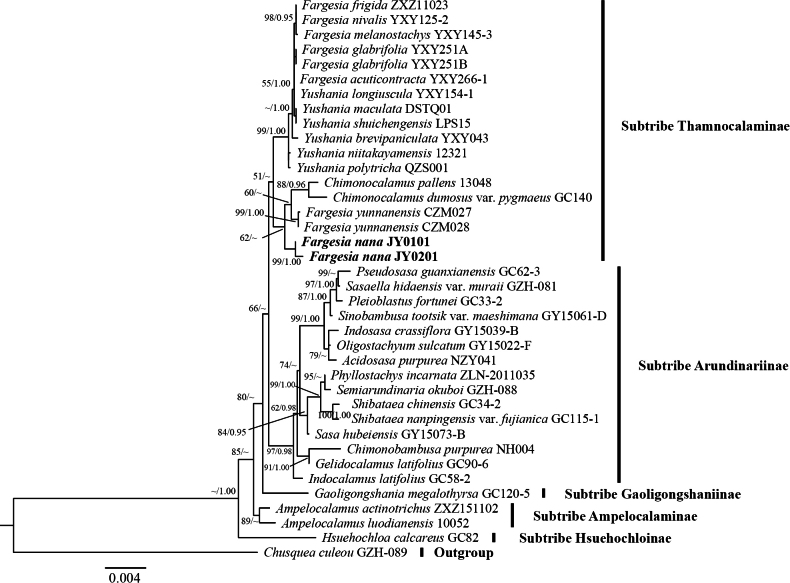
Phylogenetic tree reconstructed from nrDNA sequences by using the Maximum Likelihood (ML) and Bayesian Inference (BI) method. Numbers along branches indicate the Maximum Likelihood bootstrap values (MLBP) and Bayesian posterior probabilities (BI). “~”: nodes with Maximum Likelihood bootstrap values (MLBP) <50% (left), Bayesian posterior probabilities (BI) <95% (right).

The length of the nrDNA sequences was 6349 bp after alignment, which contained 147 variable sites and 75 parsimony information sites. In the nrDNA phylogenetic tree, five subtribes were recovered, i.e. subtribes Thamnocalaminae, Arundinariinae, Gaoligongshaniinae, Ampelocalaminae, and Hsuehochloinae. All the individuals of the new species were nested in subtribe Thamnocalaminae and clustered as a separate clade (MLBP/BI = 99/1.00) (Fig. 2).

## ﻿Taxonomy

### ﻿ Fargesia nana

Taxon classificationPlantaePoalesPoaceae

F.Du, M.Shi & J.W.Li sp. nov.

12E06307-4F47-5B19-B457-0B650F8996B8

urn:lsid:ipni.org:names:77363184-1

Figs 3 , 4

#### Diagnosis.

*Fargesianana* is morphologically similar to *F.fractiflexa* and *Yushaniapolytricha*, but can be easily distinguished by the shortened culm neck (4~8 cm vs 13~40 cm in *Y.polytricha*), branch complement (10~20(30) vs. 1~5 in *Y.polytricha*), culm leaf sheath / internode (1/3–1/2 vs. longer than internodes in *F.fractiflexa*, vs. 2/3 in *Y.polytricha*), culm blade size (0.4~2 cm × 0.8~1.7 cm vs. 2~8 cm × 1~1.5 mm in *F.fractiflexa*), auricle (absent vs. falcate auricle in *F.fractiflexa*), foliage leaf size(5~7 cm vs. 7~13 cm in *F.fractiflexa*, vs. 9~21 cm in *Y.polytricha*), foliage leaf sheath length (1.8~2.2 cm vs. 5.5~10 cm in *Y.polytricha*), transverse veins on foliage leaf blade (conspicuous vs obscure in *F.fractiflexa* and *Y.polytricha*).

**Figure 3.**
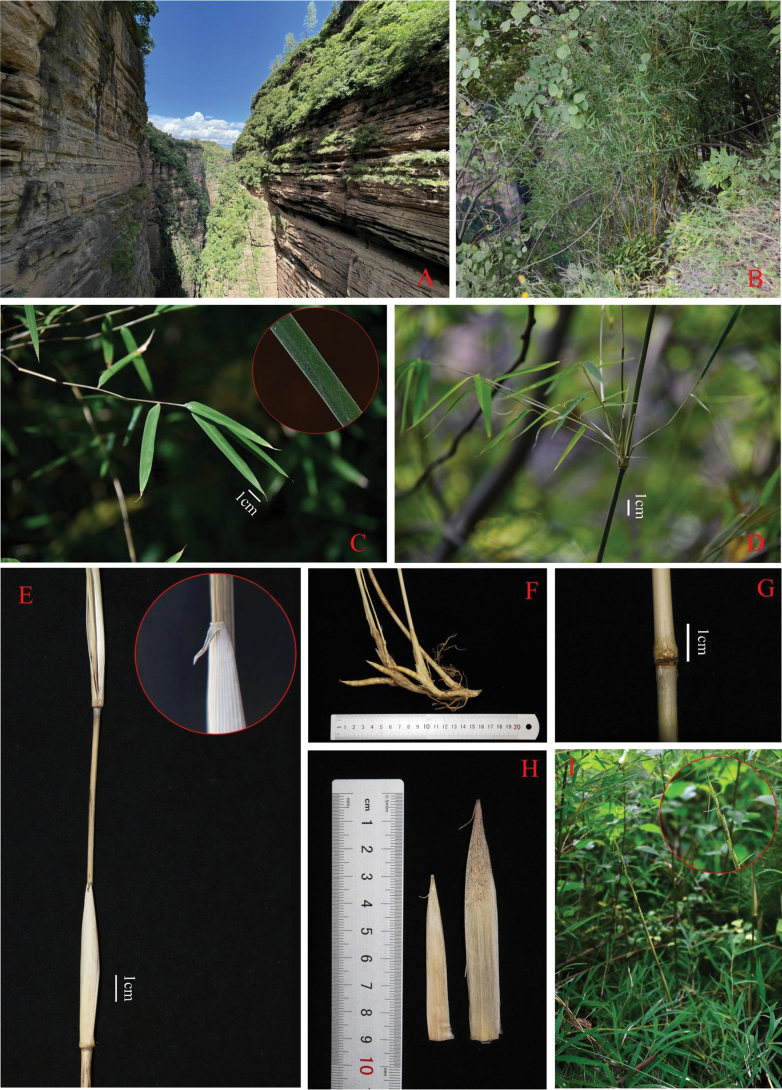
*Fargesianana* F. Du, M. Shi & J. W. Li **A** habitat **B** habit **C** leafy branch **D** branches **E** culm leaf, showing details of blade and ligule **F** rhizome **G** culm bud **H** culm leaves **I** new shoot.

#### Type.

China • Yunna: Chuxiong City, Wuding County, Jiyi Town, Jiyi Rift valley, 26°5'25.9"N, 102°15'52.7"E, 1916 m alt. 28 September 2021, *M. Shi et al. 0072410* (Holotype: SWFC!), *0072411* (Isotype: SWFC!).

**Figure 4.**
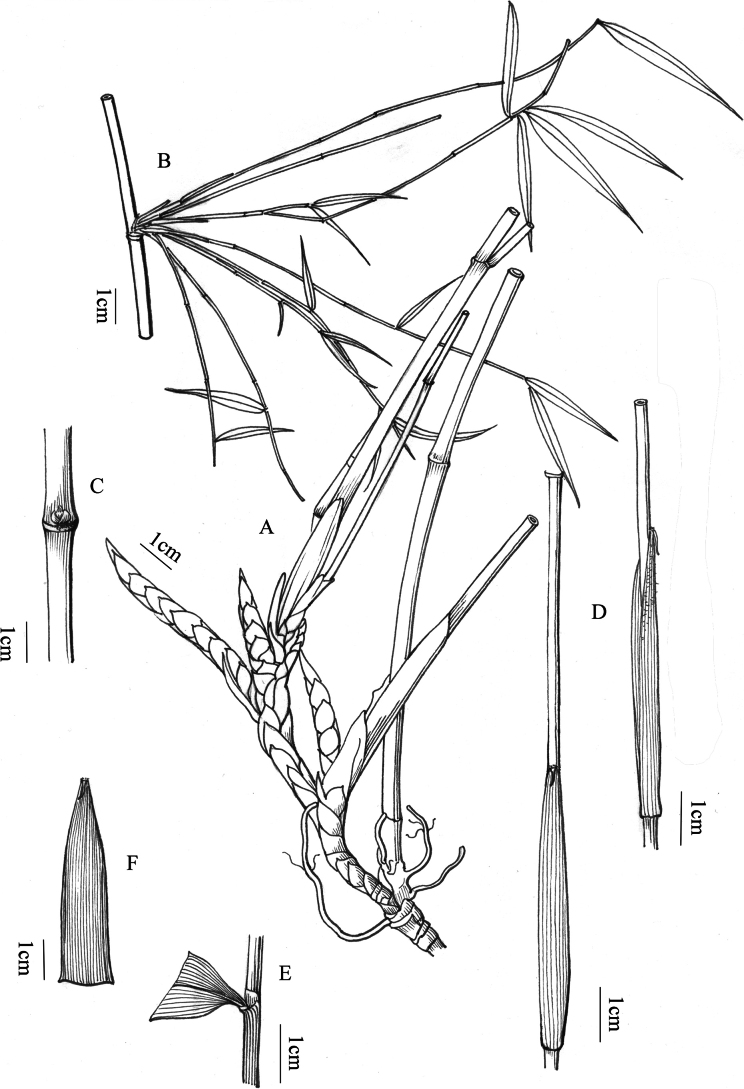
*Fargesianana* F. Du, M. Shi & J. W. Li **A** rhizome **B** branches **C** culm bud **D** part of culm, showing culm leaf **E** foliage leaf sheath and ligule **F** culm leaf.

#### Description.

Rhizomes pachymorph, neck 4–8 cm long and 0.6–0.8 cm in diameter; internodes 3–5 mm long. Culms 1–1.5 (2.2) m tall, erect, sympodial, 2–4 (6) mm in diameter; culm wall 1–2 (3) mm thick, cavity small or solid; culm with 10–15 (18) nodes, internodes terete, 10–19 (22) cm long, glabrous, without white powder and longitudinal ribs; supranodal ridge more prominent than the sheath scar, intranodes 2 mm tall. Culm buds 5–8, without obvious main buds, aggregated into a semi-orbicular composite bud. Culm branches 10–20(30), with slender secondary branches, nearly equal, less than 1 mm in diam., 20–40 cm long. Culm leaf sheaths 5–11 cm long, 1/3 to 1/2 as long as internodes, caducous, papery, light yellow-brown, narrow triangular, abaxial surface sparsely brown bristled on upper part, glabrous below; auricles and oral setae absent; ligule ca. 2 mm tall, 2–3 mm wide, truncate or lacerated at apex; blade 0.4–2 cm long, 0.8–1.7 cm wide, reflexed, linear-triangular. Foliage leaves 4–6 per ultimate branch; sheath 1.8–2.2 cm long, with obvious longitudinal veins, margins without cilia; auricles and oral setae absent; ligule truncate, ca. 1 mm tall, glabrous; petiole 2–3 mm long; blade narrowly lanceolate, 5–7 cm long and 5–7.5 mm wide, apex long acuminate, base cuneate, sparsely gray-pilose adaxially when young, glabrescent, secondary veins (2) 3 pairs, transverse veins conspicuous. Inflorescence and caryopsis unknown.

#### Phenology.

New shoots August to September.

#### Distribution and habitat.

*Fargesianana* was only found in Wuding County, Central Yunnan, China. It grows frequently in relatively humid habitats on steep cliffs at an altitude of 1895–1926 m, while coexisting with other herbs and bushes, such as *Paradombeyasinensis* Dunn, *Sunhangiayunnanensis* (Franch.) H. Ohashi & K. Ohashi, *Heteropogoncontortus* (L.) P. Beauv. ex Roemer & Schult. *Cymbopogondistans* (Nees ex Steud.) Will. Watson, *Dalbergiayunnanensis* Franch.

#### Etymology.

The specific epithet “nana” refers to the morphological characteristics of bamboo species and represents the smallest bamboo species.

#### Chinese name.

Jǐ Yī Jiàn Zhú (Chinese pronunciation); 己衣箭竹 (Chinese name).

#### Conservation status.

*Fargesianana* is an extremely rare species, and we only found three small populations in the Jiyi Rift Valley, with a total population of about 50–100 individuals. According to the IUCN Red List Categories and Criteria (IUCN 2024), it meets the criteria to be listed as Critically Endangered (CR A2acde; B2ab; C2a(i)). Given the extremely dry habitat, this new species can only survive in small, relatively moist environments within the canyon. Furthermore, *Fargesianana* is the smallest known species of *Fargesia*, possessing significant conservation and scientific research value.

#### Additional specimens examined (paratypes).

China • Yunnan: Wuding County, Jiyi Town, Jiyi Rift valley, 26°5'25.0"N, 102°15'54.1"E, 1916 m alt. 28 September 2021, M. Shi et al. 0072412 (SWFC!).

## ﻿Discussion

In “Flora Reipublicae Popularis Sinicae”, *Fargesia* was divided into two sections, i. e. section Ampullares Yi and section Fargesia (Keng and Wang 1996), based on the morphological characteristics of culm bud. The former is characterized by having semicircular, ovate or conical culm buds, consisting of several distinct buds or even multiple buds forming a composite bud that does not grow immediately against the culm, and the culm sheath is caducous, whereas the latter one is characterized by having long ovate, thin and flattened buds, consisting of a composite bud of inconspicuous several buds, closing to the stem, and the culm sheath is persistent. The new species *F.nana* has semi-orbicular composite buds, without obvious main buds, 10–20(30) culm branches with slender secondary branches, and caducous culm leaves. Therefore, we place *F.nana* into section Ampullares.

In this study, the phylogenetic trees reconstructed from plastome and nrDNA sequences revealed that the individuals of the new species clustered into two clades in the plastid tree (Fig. 1), whereas they formed a single clade with high support (100%) in the nrDNA tree (Fig. 2). Lv et al. (2023) found in their DNA barcoding study of *Fargesia* that nrDNA sequences exhibited better resolution compared to plastome sequences. Some species that appeared as polyphyletic in the plastome tree were resolved as monophyletic in the nrDNA phylogeny. Ye et al. (2021) revealed significant discrepancies between the phylogenetic relationships inferred from nuclear gene trees and chloroplast gene trees through comparative analyses of chloroplast genomes in *Fargesia*. Specifically, nuclear-based phylogeny could generally be concordant with taxonomic boundaries, whereas the plastome-based phylogeny could reflect other patterns. These findings are consistent with observations in the current study, wherein two individuals of the new species form a single clade in the nrDNA phylogenetic tree, supporting their monophyly through nuclear genomic data and further validating the reliability of nuclear gene trees in taxonomic classification. We constructed phylogenetic trees based on plastid genomes (plastome) and nuclear ribosomal DNA (nrDNA) respectively, with node support values labeled as maximum likelihood (ML, left; Bootstrap support) and Bayesian inference (BI, right; posterior probability) (Figs 1, 2). The results revealed highly congruent topological structures between the two trees, with key nodes demonstrating exceptionally strong statistical support (plastome: Bootstrap = 100%, posterior probability = 1.00; nrDNA: Bootstrap = 99%, posterior probability = 1.00). This outcome indicates that despite differences in data sources (plastid vs. nuclear genes) and analytical methods (ML vs. BI), the core framework of the phylogenetic relationships exhibits high robustness. This significantly reduces the risk of topological conflicts caused by variations in gene evolutionary rates or algorithmic biases, thereby providing reliable evidence for understanding the evolutionary history among taxa.

Furthermore, this study constructed phylogenetic trees for coding sequences (CDS) and intergenic spacers (IGS) using Maximum Likelihood (ML) and Bayesian Inference (BI) methods (Suppl. material 1), respectively. These were then compared with the plastid genome phylogeny through topological analyses (Fig. 1) to evaluate whether different datasets could generate congruent phylogenetic relationships. The results demonstrated that all three phylogenies consistently resolved the two individuals of the new species into two distinct monophyletic clades, indicating high concordance among datasets from protein-coding regions, non-coding intergenic regions, and the plastid genome (after removing one of the inverted repeats (IRs)) in phylogenetic reconstruction.

*Fargesianana* is the smallest known individual bamboo species of *Fargesia* and is restricted to the Jiyi Grand Canyon in northern Wuding County, at an elevation of 1895–1926 m. The local environment is extremely arid and no longer suitable for the large-scale distribution of bamboo species. The new species persists exclusively in small, relatively humid microhabitats within the canyon. It is a bamboo species that has adapted to the unique environment of the canyon after long-term evolutionary isolation from the external environment, reflecting the ecological preference of bamboo plants for moist conditions. The discovery of this new species may provide insights into the historical transition of the climate in central Yunnan, from humid to increasingly arid conditions. Such climatic changes have likely caused moisture-preferred species to retreat into relatively humid microhabitats within canyons. The impact of these climatic shifts on plant species and vegetation distribution in central Yunnan warrants further investigation. Additionally, this discovery highlights the possibility of more undiscovered species persisting in the isolated and humid environments of central Yunnan, such as deep canyons, sinkholes, or enclosed river valleys. These findings have significant implications for further exploration and conservation of the species diversity in this region.

## Supplementary Material

XML Treatment for Fargesia nana

## ﻿Additional information

### Author ORCIDs

Jian-Wei Li https://orcid.org/0009-0004-5362-982X

Juan Wang https://orcid.org/0009-0008-6762-9627

Ming Shi https://orcid.org/0009-0009-7767-0508

Fan Du https://orcid.org/0000-0003-1074-8891

### Data availability

All of the data that support the findings of this study are available in the main text or Supplementary Information.

## Supplementary materials

Supplementary material 1 Supplementary figures Data type docx Explanation note **figure S1.** Consensus phylogenetic tree reconstructed by Maximum Likelihood (ML) and Bayesian Inference (BI) analysis based on intergenic spacers (IGS) sequences data. **figure S2.** Phylogenetic tree of *Fargesianana* and related species based on coding sequences (CDS) sequences by using the Maximum Likelihood (ML) and Bayesian Inference (BI) method. https://binary.pensoft.net/file/1350156This dataset is made available under the Open Database License (http://opendatacommons.org/licenses/odbl/1.0/). The Open Database License (ODbL) is a license agreement intended to allow users to freely share, modify, and use this Dataset while maintaining this same freedom for others, provided that the original source and author(s) are credited.Jian-Wei Li, Juan Wang, Ming Shi, Fan Du
