# Supplementary material for: ﻿Fargesianana (Poaceae, Bambusoideae), a new bamboo species from Yunnan, China
Source: PhytoKeys. 2025 Jun 11;257:219–33. doi: 10.3897/phytokeys.257.154092 (PMC12177521; doi:10.3897/phytokeys.257.154092)
Supplement: Supplementary material 1 — Supplementary figures [file phytokeys-257-219_article-154092__-s001.docx]

**Supplementary Files**

**Title: *Fargesia nana* (Poaceae, Bambusoideae), a new bamboo species from Yunnan, China**

Figure S1. Consensus phylogenetic tree reconstructed by Maximum Likelihood (ML) and Bayesian Inference (BI) analysis based on intergenic spacers (IGS) sequences data.

Figure S2. Phylogenetic tree of *Fargesia nana* and related species based on coding sequences (CDS) sequences by using the Maximum Likelihood (ML) and Bayesian Inference (BI) method.


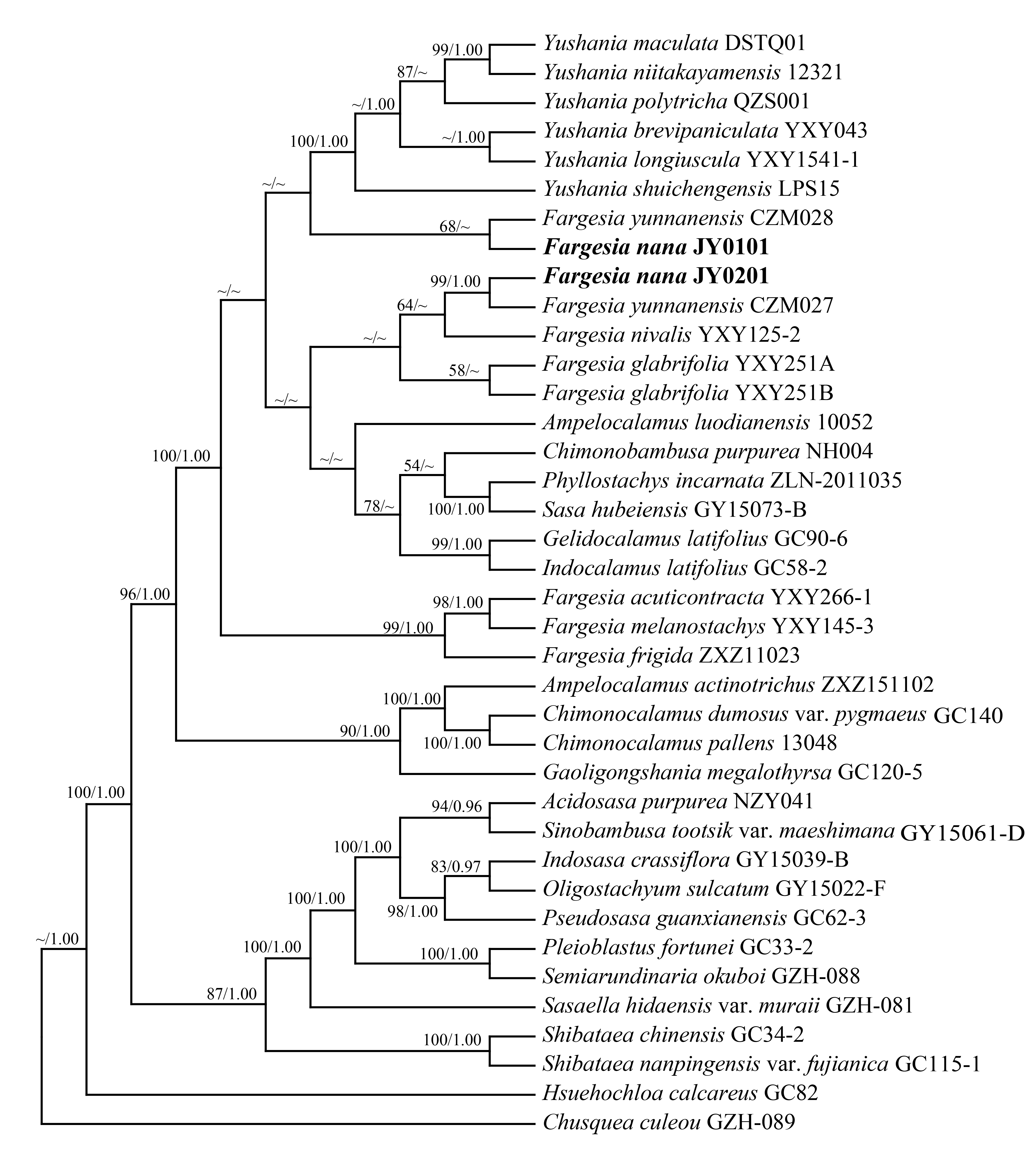


Figure S1. Consensus phylogenetic tree reconstructed by Maximum Likelihood (ML) and Bayesian Inference (BI) analysis based on intergenic spacers (IGS) sequences data. Numbers along branches indicate the Maximum Likelihood bootstrap values (MLBP) and Bayesian posterior probabilities (BI). “~”: nodes with Maximum Likelihood bootstrap values (MLBP) <50% (left), Bayesian posterior probabilities (BI) <95% (right).


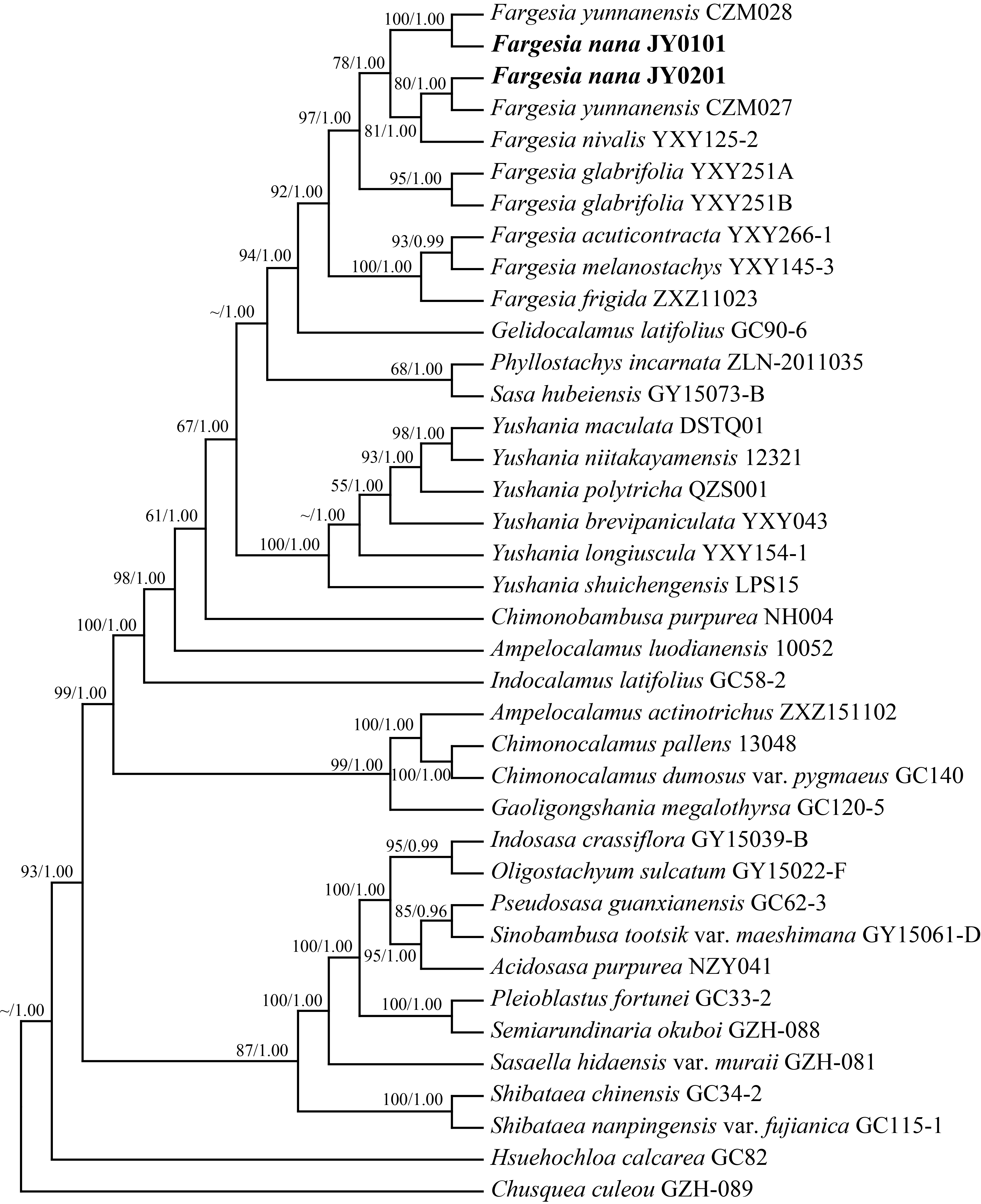


Figure S2. Phylogenetic tree of *Fargesia nana* and related species based on coding sequences (CDS) sequences by using the Maximum Likelihood (ML) and Bayesian Inference (BI) method. Numbers along branches indicate the Maximum Likelihood bootstrap values (MLBP) and Bayesian posterior probabilities (BI). “~”: nodes with Maximum Likelihood bootstrap values (MLBP) <50% (left), Bayesian posterior probabilities (BI) <95% (right).
